# Advances in Nanomaterials in Biomedicine

**DOI:** 10.3390/nano11010118

**Published:** 2021-01-07

**Authors:** Elena Ryabchikova

**Affiliations:** Institute of Chemical Biology and Fundamental Medicine, Siberian Branch of Russian Academy of Science, 8 Lavrentiev Ave., 630090 Novosibirsk, Russia; lenryab@yandex.ru

**Keywords:** nanotechnology, nanomedicine, biocompatible nanomaterials, diagnostics, nanocarriers, targeted drug delivery, tissue engineering

Biomedicine is actively developing a methodological network that brings together biological research and its medical applications. Biomedicine, in fact, is at the front flank of the creation of the latest technologies for various fields in medicine, and, obviously, nanotechnologies occupy an important place at this flank. Based on the well-known breadth of the concept of “Biomedicine”, the boundaries of the Special Issue “Advances in Nanomaterials in Biomedicine” were not limited, and authors could present their work from various fields of nanotechnology, as well as new methods and nanomaterials intended for medical applications. This approach made it possible to make public not only specific developments, but also served as a kind of mirror reflecting the most active interest of researchers in a particular field of application of nanotechnology in biomedicine. The Special Issue brought together more than 110 authors from different countries, who submitted 11 original research articles and 7 reviews, and conveyed their vision of the problems of nanomaterials in biomedicine to the readers.

A detailed and well-illustrated review on the main problems of nanomedicine in onco-immunotherapy was presented by Acebes-Fernández and co-authors [1]. It should be noted that the review is not limited to onco-immunotherapy, and gives a complete understanding of nanomedicine in general, which is useful for those new to this field. The review is well structured, and clear schemes help the reader to follow complicated problems of cancer diagnostics and treatment using nanomedicine approaches.

The review authored by Bromma and Chithrani [2] critically discusses the state of the art problems and prospects for using AuNPs (gold nanoparticles) in combined cancer therapy. The authors presented variants of nanoplatforms based on AuNPs and the role of their physicochemical characteristics in determining the pathways of interaction with cells, and they draw attention to the problem of NPs biocompatibility for their successful application. This review analyzes the use of AuNPs as radiosensitizers and in chemoradiotherapy, highlighting problems and outlining prospects for the development of existing applications. The use of multicellular spheroids as adequate experimental tumor models is mentioned in this review, and the article presented by Chelobanov and co-authors [3] provides new data on spheroids formed by human hepatoma cells (Hep-G2) and human kidney cells (HEK293). The authors show the preservation of organ specificity in the structure of HepG2 spheroids, whose cells form bile capillaries and “pseudosinusoids”. For researchers working with spheroids, it will be interesting to know that the outer surface of the spheroids, which is in contact with the nutrient medium, is formed by the basal regions of cells. This work also reported the inability of HepG2 cells to internalize “naked” AuNPs in both monolayer and spheroids, in contrast with active uptake of the same AuNPs covered with polyethylenimine (PEI) or bovine serum albumin (BSA). The HEK293 cells were devoid of this “selectivity” and internalized all types of AuNPs.

AuNPs possess many unique physico-chemical properties and are widely used in various biomedicine studies, mostly in spherical form, which can be easily synthesized and functionalized. D’Hollander and co-authors [4] reported obtaining and characterizing gold nanostars, and their high potential as agents of theranostics. This paper presents the successful use of gold nanostars that were coated with a polyethyleneglycol-maleimide for in vitro and in vivo photoacoustic imaging, computed tomography, as well as photothermal therapy of cancer cells and tumor masses. The work was carried out using a wide range of methods; however, an understandable presentation of experiments will allow other researchers to reproduce the obtaining of nanostars and conduct studies with them. The experiments performed by the authors convincingly demonstrate the high potential of the nanostars they obtained for theranostics and clinical use.

Iron NPs are another widely used object in biomedicine, and the Special Issue has combined their research in a variety of areas, reflecting the interest of a broad audience in this object. Thus, Veloso and co-authors [5] shared their experience in obtaining a new nanoform, representing a combination of solid and aqueous magnetoliposomes with supramolecular peptide-based hydrogels. Such complex constructions are primarily intended for the safe and targeted delivery of toxic drugs that are used in cancer therapy, and the authors provided evidence for their architecture and spatial organization.

Luo and co-authors [6] presented a new level of iron NPs application. Using RNA sequencing, the ability of Fe_3_O_4_ NPs coated with 2,3-Dimercaptosuccinic acid (DMSA) to induce the production of reactive oxygen species (ROS), as well as the ability of Prussian blue NPs to be ROS scavengers, was shown on two leukemia cell lines (KG1a and HL60). This research is part of a promising new approach to the treatment of leukemia—the development of nanomedical drugs as inducers of ferroptosis in cancer cells. The authors applied modern methods of transcriptome analysis and showed that two types of iron NPs differently regulate many genes expression. Surprisingly, it turned out that NP treatment of cells could affect the expression of many important genes, including those associated with antioxidant effects, lipid metabolism, vesicle movement, innate immune system, and the cytoskeleton. The importance of this work lies not only in its elucidation of iron NPs’ cytotoxicity mechanisms for leukemic cells, but also in demonstration that NPs could affect the molecular mechanisms of cell metabolism. Obviously, the time has come to study the effects of NPs on cells and the organism at the molecular level, not limited to standard cytotoxicity tests.

Thus, the effect of NPs can lead to the disruption of cellular intermolecular interactions, in particular, to enhance the processes of peroxidation, which play an important role in stress development. However, it is well known that a poison can be a medicine, and vice versa, and Kumar with co-authors in their paper [7] reviewed the possibility of using various nanomaterials as anti-stress agents. Having shown the role of peroxide reactions in the development of various diseases, the authors move on to the analysis of antioxidants and the possible use of NPs, including metallic, to combat oxidative stress. They present information on the antioxidant properties of a number of NPs, as well as on the use of NPs as carriers of antioxidants and on the functionalization of NPs with antioxidants. The use of nanomaterials as antioxidants is a new direction in nanomedicine, and this review gives a comprehensive understanding of the state of research in this area. The text provides detailed characteristics of nanomaterials that have shown antioxidant properties and discusses the problems of their implementation into practice.

The Special Issue includes a review on the use of nanomedicines in the diagnosis and treatment of liver fibrosis [8], which remains among the difficult-to-diagnose and poorly treatable diseases. Xue Bai and co-authors inform readers about the crucial role of hepatic stellate cells in pathogenesis of liver fibrosis and briefly summarize routine approaches for the treatment and diagnostics of this severe disease. Then, data on use of magnetic NPs in magnetic resonance imaging for fibrosis diagnostics are presented. Analyzing the application of different nanomaterials for the treatment of liver fibrosis, the authors pay attention to the positive therapeutic effects of metallic NPs, drug delivery by various NPs and the use of targeted ligands to improve drug delivery. To complete the review, the authors show the advantages and disadvantages of using nanomedicines for liver fibrosis treatment and diagnosis, and express their confidence that all the imperfections of nanomedicine could be overcome by the design and fabrication of nanosystems based on knowledge about their properties.

The paper submitted by Köse and co-authors [9] presents molecular ultrasound imaging basing on nanoconstructions, such as microbubbles, nanobubbles, and nanodroplets, which are able to significantly improve standard ultrasound examinations. This paper demonstrates the advantages of new imaging systems in the visualization of blood vessels in particular, which is an important task for many clinical examinations. The authors emphasize that the presented means of molecular ultrasound diagnostics have been tested in laboratory models and, in fact, are at the level of preclinical studies. They focus on the “painful” problem of translating new developments into the clinic, which requires appropriate funding. This issue has also been noted in other articles in the Special Issue, and it is hoped that this podium will better demonstrate the potential of new developments in nanomedicine and attract the attention of potential investors.

The Special Issue “Advances in Nanomaterials in Biomedicine”, among others, announced the topic “New Effective Antimicrobials”. Khalir and co-authors [10] presented a process of preparing Ag-NPs on alkali-treated *C. pentandra* fibers as supporting material, and also provided detailed characteristics of the resulting products. The *C. pentandra*/Ag-NPs showed catalytic activity towards the Rhodamine B and methylene blue dyes, and good antibacterial activity towards both Gram-positive and Gram-negative bacteria *S. aureus*, *E. faecalis*, *E. coli* and *P. vulgaris*. Thus, *C. pentandra*/Ag-NPs could be evaluated as a promising nanomaterial for biomedical applications such as wound healing and the coating of biomaterials, wastewater treatment, food packaging, etc.

The growing use of nanomaterials is a cause for concern, as many of them have negative effects on living organisms. The assessment of this effect is often complicated by the presence of one or another element in biogenic forms in the body, which raises the problem of distinguishing between a biogenic and an element originating from nanomaterials. The article by Škrátek and co-authors [11] shows how it is possible to detect and differentiate iron, which comes from nanomaterials, and iron, which is naturally present in living organisms. To this end, the authors improved the SQUID magnetometric method, and showed that the developed method makes it possible to detect “artificial” iron in solid and liquid samples of animal tissues, and to distinguish it from biogenic iron naturally present in tissues and blood.

The creation of ever new nanomaterials and the study of the possibility of their application in biomedicine gave rise to the need for the analysis of published data in accordance with individual branches of medicine. The review presented by Kumar and co-authors [12] summarizes the data on prospects for the use of nanomaterials in the field of solid tissue engineering and analyzes the types of nanomaterials suitable for this particular area. The main tasks that can be solved using nanomaterials are shown, including the construction of scaffolds that mimic the extracellular matrix; regeneration of bones and cartilage; fighting infection through the properties of nanomaterials

The problems of using nanomaterials for drug delivery attract the attention of many researchers, and it is not surprising that five original articles and one review in the Special Issue are devoted to the development of delivery vehicles, based on various types of nanomaterials. A review by Idrees and co-authors [13] critically examines the use of naturally occurring biodegradable polymers in drug delivery systems for local or targeted and controlled release of drugs. The authors dwell in detail on the most used biodegradable and biocompatible substances, such as chitosan, alginate, albumin, hyaluronic acid and hydroxyapatite, giving not only their main characteristics, but also analyzing their advantages and disadvantages. This review will be useful to novice researchers, who can gain comprehensive information on the state of art in the field of drug delivery using biodegradable delivery vehicles.

Meng and co-authors [14] demonstrated a new approach to the treatment of articular cartilage injuries using a nanoconstruct based on selected carbon dots coupled to a recombinant adeno-associated virus (rAAV) vector for the delivery of highly chondroreparative cartilage-specific sex-determining region Y-type high-mobility group 9 (SOX9) transcription factor or transforming growth factor beta to human bone marrow-derived mesenchymal stromal cells. All experiments are detailed in this article, and the data presented convincingly demonstrates that the selected carbon dots are systems for efficient gene transfer through rAAV. Many people suffer from osteoarthritis, which is associated with irreversible degradation of key components of the articular extracellular cartilage matrix (ECM) (proteoglycans, type II collagen), with the critical involvement of pro-inflammatory cytokines (interleukin 1 beta and tumor necrosis factor alpha). Urich and co-authors [15] reported effective, micelle-guided rAAV SOX9 overexpression, which enhanced the deposition of ECM components and the level of cell survival. In other words, the operation of this rAAV vector system provided the neutralization of negatively acting cartilage cytokines, and polymer micelles, in turn, provided its controlled delivery, thereby evidencing the significance of appropriate nanomaterial development.

The efficient delivery of therapeutic nucleic acids into cells is one of the most important problems, and numerous and diverse nanoconstructions are being created to solve it. In the Special Issue, in addition to carbon dots and polymer micelles, two more types of carriers are presented: dodecyl-containing oligonucleotides [16] and nanosystems based on tyrosine-modified polypropylenimine (PPI) and polyethylenimine (PEI) [17]. In the first, Pavlova and co-authors [16] showed the formation of micellar nanostructures by the self-assembly of amphiphilic “like-a-brush” oligonucleotide conjugates, and their penetration into HepG2 cells. This is the first report on the self-assembly of “like-a-brush” triple chains-contained dodecyl-bearing oligonucleotides and their ability to enter the cells; thereby, this work widened a set of means for nucleic acids delivery. The authors of the second article exploit chemical modification to improve siRNA delivery with well-known cationic polymers, namely polyethylenimine (PEI) and polypropylenimine (PPI). Noske and co-authors [17] for the first time modified PPI, including dendrimer form, by tyrosine and showed that this modification significantly improved the siRNA complexation, complex stability, siRNA delivery, knockdown efficacy and biocompatibility. It should be noted that analysis of the effect of corresponding siRNAs on cytokines TNF-α and INF-γ was carried out in mice, which makes conclusions about the effect of tyrosine modification more convincing. As a whole, this work demonstrated that tyrosine-modified PPIs or PEIs are promising polymeric systems for siRNA formulation and delivery.

An example of the self-assembly of nanoconstructions for drug delivery has been given in the paper by Apartsin and co-authors [18], who designed a new class of dendritic amphiphiles self-assembling into vesicle-like supramolecular associates (dendrimersomes). The rationally designed molecular topology of dendrons permits us to use simple procedures to yield monodisperse pH-sensitive NPs. As a proof-of-concept study, anti-cancer drugs doxorubicin and methotrexate were encapsulated into dendrimersomes and delivered into human leukemia cells. Drug-loaded dendrimersomes efficiently penetrate into cells and induce cell death.
